# Three-Dimensional Phenotyping Pipeline of Potted Plants Based on Neural Radiation Fields and Path Segmentation

**DOI:** 10.3390/plants13233368

**Published:** 2024-11-29

**Authors:** Xinghui Zhu, Zhongrui Huang, Bin Li

**Affiliations:** 1 College of Information and Intelligence, Hunan Agricultural University, Changsha 410128, China; zhuxh@hunau.edu.cn; 2 Hunan Engineering Technology Research Center of Agricultural Rural Informatization, Changsha 410128, China; hhhzzr542711013@163.com

**Keywords:** 3D reconstruction, stem and leaf segmentation, neural radiance fields, structure from motion, potted plant analysis, plant digitization, neural network, phenotypic calculation

## Abstract

Precise acquisition of potted plant traits has great theoretical significance and practical value for variety selection and guiding scientific cultivation practices. Although phenotypic analysis using two dimensional(2D) digital images is simple and efficient, leaf occlusion reduces the available phenotype information. To address the current challenge of acquiring sufficient non-destructive information from living potted plants, we proposed a three dimensional (3D) phenotyping pipeline that combines neural radiation field reconstruction with path analysis. An indoor collection system was constructed to obtain multi-view image sequences of potted plants. The structure from motion and neural radiance fields (SFM-NeRF) algorithm was then utilized to reconstruct 3D point clouds, which were subsequently denoised and calibrated. Geometric-feature-based path analysis was employed to separate stems from leaves, and density clustering methods were applied to segment the canopy leaves. Phenotypic parameters of potted plant organs were extracted, including height, stem thickness, leaf length, leaf width, and leaf area, and they were manually measured to obtain the true values. The results showed that the coefficient of determination (R^2^) values, indicating the correlation between the model traits and the true traits, ranged from 0.89 to 0.98, indicating a strong correlation. The reconstruction quality was good. Additionally, 22 potted plants were selected for exploratory experiments. The results indicated that the method was capable of reconstructing plants of various varieties, and the experiments identified key conditions essential for successful reconstruction. In summary, this study developed a low-cost and robust 3D phenotyping pipeline for the phenotype analysis of potted plants. This proposed pipeline not only meets daily production requirements but also advances the field of phenotype calculation for potted plants.

## 1. Introduction

With people’s demand for a quality living environment increasing, potted plants have become increasingly popular in recent years, as they have been shown to improve indoor air quality (IAQ) [1], reduce environmental carbon dioxide levels, and increase water content through photosynthesis and evapotranspiration. Potted plant organs and their characteristics are very important to the physiological status of many potted plants [2]. Phenotyping can assist in breeding, thereby improving yield and quality [3]. Selecting potted plants as test objects is beneficial for simulating ideal growth conditions and saving costs.

Traditional methods of phenotypic information acquisition involve in-field data collection with hand-held tools, which is time-consuming and laborious, often damaging the potted plant’s structure, as well as inefficient and lacking in objective consistency [4,5,6]. The question of how to obtain all-around information on living potted plants in a non-destructive and accurate way [7] has become a significant topic. Various phenotyping technologies have gained much attention in the agricultural field because of the rapid development of new sensors and corresponding automation technology. Many methods are based on 2D digital images and focus on the defects, colors, and shapes of potted plants [8]. Tian Yvfeng et al. [9] extracted leaf regions of *Cucurbita pepo* L. seedlings by threshold segmentation and used a method for segmenting the images based on maximum inter-class variance, and geometric features were used to determine the direction of leaf growth. Zhang Derong et al. [10] developed a method based on the hue, saturation, and value (HSV) image eigenvalue, which accomplished accurate measurements of leaf area and detection of leaf nitrogen content using color parameters. Zhang Xunmeng et al. [11], based on the YOLOv3 neural network model, extracted the fruit component of the *Solanum lycopersicum* L. pink crown F1. The phenotypic parameters of the length, width, and projected area of *Solanum lycopersicum* L. fruits were calculated by a support vector machine (SVM), with an average relative error of 6.45%. To identify the outline of *Lactuca sativa*, Li Xiuhuaet al. [12] proposed a robust method for image edge segmentation and used it to identify regions. The results showed a significant linear correlation between the calculated phenotypic parameters, with an average coefficient of determination of 0.91%. Hu Lingyan et al. [13] combined a time-overlapping algorithm and morphological threshold filtering to predict the key regions of the surface of the *Prunus avium* L. This algorithm performed well under complex scenarios, with an intersection over union (IoU) of 0.91%.

The aforementioned techniques mainly used digital 2D images [14,15], due to characteristics such as spatial self-occlusion of the potted plants, reducing the amount of information available [16]. On the other hand, image-based techniques often require operator training [17]. In recent years, with the increasing accuracy of potted plant phenotypic measurements and the popularization of 3D sensors such as laser scanners, light detection and ranging(lidar), and time of flight(ToF )cameras [18], more attempts have been made to study phenotypes based on 3D information, and 3D phenotypic techniques have gradually become a powerful tool for obtaining phenotypic traits, due to their noninvasive and non-contact properties.

**Figure 1:**
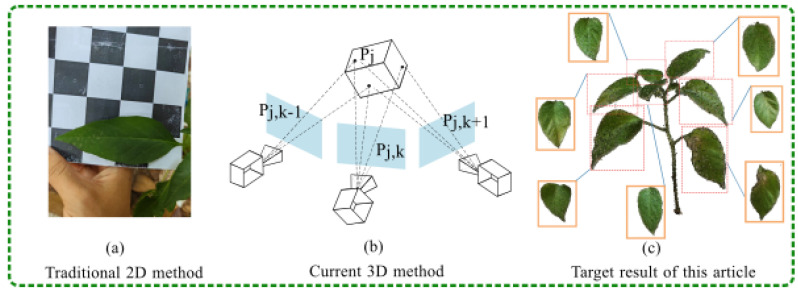
Manual acquisition of phenotypic data through traditional methods is not only inefficient and labor-intensive but also poses a risk of damage to potted plants. Furthermore, 2D technologies are impeded by plant occlusion, and the costs associated with current 3D methodologies remain high. Consequently, this study aimed to employ a neural-radiance-field-based approach to swiftly and accurately ascertain phenotypic information from potted plants in a non-destructive manner.

The schematic diagrams of the traditional two-dimensional and classical three-dimensional methods are shown in Figure 1. Xie Weijun et al. [19] used a depth camera to obtain multiview images of *Daucus carota* L., 3D reconstructed the *Daucus carota* L. using Poisson model reconstruction and inverse projection methods, and manually measured the volume and length, and these measurements were compared with the reconstructed results, with mean absolute percentage errors (MAPE) of less than 3%. Sun Guoxiang et al. [20] used a depth camera to capture point cloud data of potted plants from multiple angles for fast and accurate measurement of shape parameters, and used an iterative closest point algorithm to achieve alignment of a multi-view point cloud, and the test results showed an average relative deviation (RAD) of 8.52%. Although the point clouds generated by the above study were effective, the calculation can be disturbed by various factors, including the external environment and the internal structure of the camera [21]. The accuracy of such depth imaging is affected by the object reflectivity, imaging distance, ambient light, etc.

The superiority of using 3D information in the calculation of phenotypic traits, such as plant volume, plant height, and leaf length, has been demonstrated. SFM and Multi-view stereo (MVS) methods are combined together to generate a dense point cloud for objects in the most common algorithm [22]. In a moving-camera scenario, Jordan Miller et al. [23] used SFM-MVS technology to reconstruct a 3D model of a single tree, and obtained 3D information such as the tree height, canopy depth, stem thickness, and volume metrics. Maria Immacolata Marzulli et al. [24] photographed *Pinus halepensis Mill.* using a digital camera in an outdoor environment and performed 3D reconstruction using the SFM method. Their research estimated structural parameters, including trunk length and width, from the 3D model representation of the plants. The results were compared with other algorithms in terms of error. The reconstruction method based on SFM-MVS requires only one or more red, green, blue(RGB) cameras to capture data for reconstruction, with a low reconstruction cost, but for potted plants with severe canopy shading, the reconstruction quality is then affected. ﻿

A lot of research has been carried out on crop phenotypes based on 3D information [25], but there has been a lack of research on potted plant. Test objects were planted in trays in the form of pots, which helped to address and understand the relation between potted plant growth and the surrounding environment, and prepared for the subsequent 3D reconstruction. In conclusion, numerous scholars have conducted observations on crops such as *Gossypium hirsutum* L. [26], *Solanum lycopersicum* L. [15], and *Glycine max* (L.) Merr. [27].

This paper addresses the issue of obtaining all-around information on living potted plants in a non-destructive and accurate way. We proposed a 3D phenotyping pipeline for potted plant reconstruction and phenotypic extraction based on NeRF and a clustering algorithm, and collected different varieties of potted plant point cloud data as datasets in both a fixed-camera scenario and a moving-camera scenario. Subsequently, the segmented *Capsicum annuum* L. organ point clouds were used to calculate various phenotypic parameters. The results showed that the measurement values of the phenotypic parameters had a strong correlation with the real values, contributing to precisely extracting and analyzing 3D phenotypic structural information of potted plants. The main contributions of this paper are as follows:

(1) We collected different varieties of potted plant images as a dataset, SFM-NeRF were combined to construct and generate 3D point cloud models of these potted plants from a series of 2D images.
(2) This article used the shortest path analysis algorithm to separate stems and leaves, and flexibly used k-nearest neighbors(KNN) and density based spatial clustering of applications with noise(DBSCAN) techniques to cluster and segment canopy leaves, achieving an accurate segmentation of occluded leaves and obtaining geometric features of potted plants, providing an efficient and accurate method for phenotype analysis of potted plants.
(3) Our calculated phenotypic parameters of segmented *Capsicum annuum* L. organs revealed a strong correlation with the actual values. This indicated the potential for our method to accurately measure key phenotypic parameters non-destructively. Further research will confirm its applicability across different species and varieties, thereby enhancing the technical support for plant breeding and cultivation studies. ﻿

## 2. Materials and Methods

### 2.1. Data Acquisition

**Figure 2:**
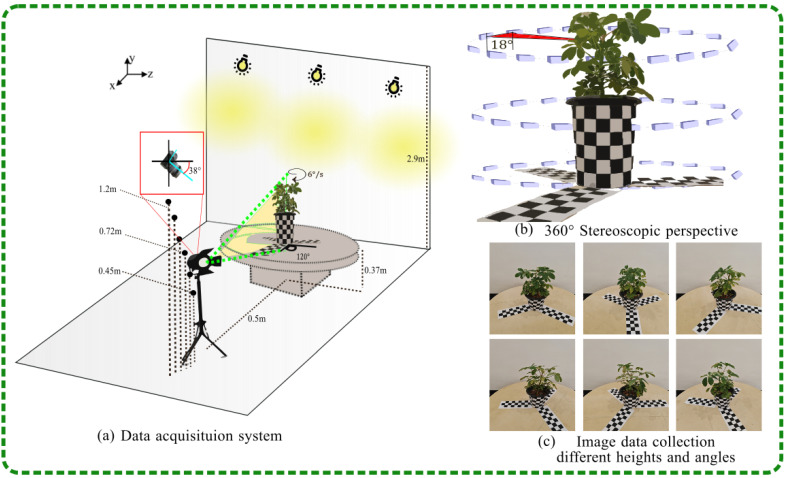
Data collection of indoor potted plants.

This study was conducted in January 2024 at the indoor Experiment Station of Hunan Agricultural University, located at longitude 113°4′ E and latitude 28°10′ N, where potted plant image collection was performed. In this study, the morphological structure of potted plants was examined in a controlled environment designed to minimize the effects of shadows and wind for accurate observation. Incandescent lamps were fixed at equal distances on a bright, pure white wall at a height of 2.9m to create a uniformly lit indoor environment. Potted plants were selected as the study subjects and photographed using a phenotypic platform. The phenology platform for greenhouse potted plants mainly consisted of a rotating platform, a tripod, and cardboard with a checkerboard pattern, as shown in Figure 2.

**Figure 3:**
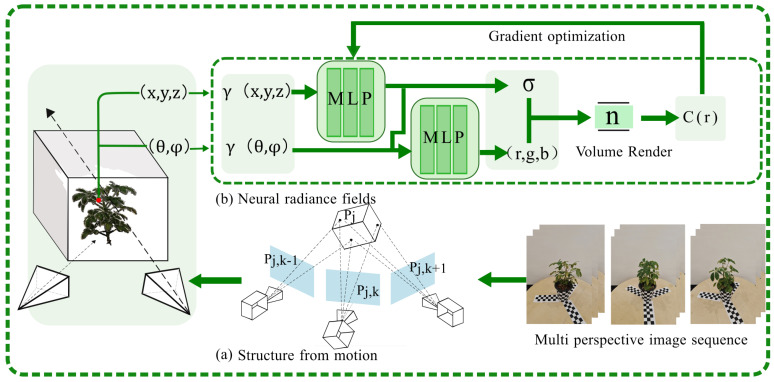
Overall flowchart of potted plant reconstruction.

As shown in Figure 2a, the rotating platform, standing at a height of 0.37 m, was positioned adjacent to the wall. The potted plant was placed at the center of the rotating platform. The camera, mounted on a tripod and angled downwards, was positioned 0.5 m away from the potted plants. This setup ensured that both the turntable and the potted plants were fully within the camera’s field of view. The rotating platform had a diameter of 0.97 m. Calibration cardboard was attached to the side of the potted plant, and three calibration cardboard strips were carefully positioned under the pots. These were aligned using the center of the rotating platform as a reference point. This setup provided a reliable reference for precise measurements and scaling when capturing and processing the point cloud data of the potted plants. As shown in Figure 3, the potted plants were rotated at a constant speed, with the turntable operating at a speed of 6 degrees per second. The camera was set to capture images at intervals of 3 s. It took images at various heights: 0.45 m, 0.62 m, 0.72 m, 0.79 m, 1.03 m, and 1.22 m. For each height, the camera captured 20 images, resulting in a total of 120 images. The acquisition scheme, which details the process of capturing the data, is described below, while the multi-view RGB images obtained as part of the study are displayed in Figure 2c. Potted plant point cloud reconstruction and point cloud segmentation were performed in Ubuntu and Python 3.9 using the open-source computer vision library Open3D (version 4.0.0, available at Open3D) and a 3D point cloud and mesh processing software CloudCompare (version 2.11.3, available at CloudCompare).

### 2.2. Reconstruction Methods

This paper presents a reconstruction algorithm that merges the strengths of traditional SFM techniques with NeRF algorithms [28]. This hybrid approach adeptly captures the characteristics of potted plants, offering a more precise and efficient methodology for analyzing potted plant phenotypes.

SFM and MVS methods were combined to create 3D models of potted plants. The SFM workflow involved several steps: (1) Feature Consistency: The process begins by searching for consistent features across all input images; (2) Key Point Descriptors: Key points are extracted from the images of the potted plant; (3) Descriptor Matching: For each descriptor, potential matching positions are estimated across the entire set of images; (4) Triangulation: Once matches have been identified, the points are triangulated to determine their 3D coordinates, transforming the 2D feature matches into a 3D point cloud; (5) Optimization: Iterations are optimized to refine the point cloud model, ensuring that the 3D model is as accurate and detailed as possible.

The feature descriptor in SFM must be distinctive across the entire scene, which typically results in a sparse point cloud model, because it focuses on representing only a small, distinctive region in the images. To address this limitation, the MVS algorithm is employed. Leveraging the camera positioning data derived from SFM, the MVS algorithm enhances the accuracy of 3D point matching. This process refines the SFM output, enabling the creation of a detailed and dense point cloud for potted plants. The MVS algorithm excels at capturing the comprehensive structure of a scene and is particularly efficient for tasks involving rapid analysis of extensive image datasets.

The NeRF approach has emerged as a significant area of focus within the realm of 3D reconstruction, garnering interest from researchers across related disciplines. This is largely due to its remarkable capabilities in delivering superior visual quality and achieving compelling view synthesis. Crop model reconstruction using NeRF, as referenced in [29], employs technology where it is hypothesized that each point within a potted plant exists within a radiative space. Within this field, every point is characterized by an aggregation of radiances emanating from various directions, which together determine the point’s transparency and color. These radiances are modeled and learned by a multilayer perceptron (MLP), to effectively predict and represent the 3D structure of the potted plant.

**Figure 4:**
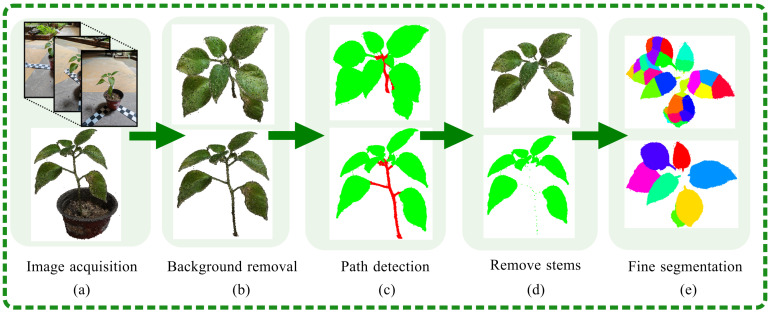
Overall flowchart of stem and leaf segmentation. The red color at the beginning represents the stem, the green color represents the leaves, and the subsequent colors correspond to the successfully segmented individual leaves.

As shown in Figure 4, the NeRF model ingests the five dimensional(5D)coordinates from the potted plant’s point cloud, which encapsulates both the 3D spatial position and the 2D viewing direction, as an input representing the viewpoint information. The MLP operates as a decoder, tasked with outputting the color and density of the pixel at the specified position; the specific formula is shown in Equation (1). This output is a prediction based on the learned radiance field. The algorithm compares the estimated pixel values from the MLP with the actual pixel values from the image data. This comparison allows the calculation of a loss function, which quantifies the difference between the predicted and real values. To refine the model, the parameters of the MLP are adjusted through a process called backpropagation. This iterative process minimizes the loss function, progressively improving the accuracy of the 3D model reconstruction. The algorithm is adept at reconstructing detailed parts of the image even with a relatively small amount of input data. This capability is particularly valuable in scenarios where data are scarce or difficult to obtain, showcasing the algorithm’s efficiency and robustness in 3D modeling tasks.

(1) $$\begin{array}{cc} \hat{C}\left(r\right) & =\sum_{i=1}^{N}T_{i}\left(1-\exp\left(-\sigma_{i}\delta_{i}\right)\right)c_{i},\\ T_{i} & =\exp\left(-\sum_{j=1}^{i-1}\sigma_{j}\delta_{j}\right). \end{array}$$

Here, $T_{i}$ represents the transmittance, which is the probability that a ray passing through the scene will not interact with any surface up to the *i*-th layer. The term $\exp\left(-\sigma_{i}\delta_{i}\right)$ models the probability of interaction with the *i*-th layer, where $\sigma_{i}$ is the density and $\delta_{i}$ is the distance traveled through the *i*-th layer. The color $c_{i}$ is then weighted by this transmittance to account for the color contribution from each layer. In this research, the paper presents a synergistic approach that harnesses the strengths of both SFM and NeRF. The SFM algorithm is employed as an initial step for coarse extraction, swiftly capturing the general structure of the scene and determining the camera’s internal parameters and positional data. This lays the groundwork for a 3D representation. Following the coarse extraction phase, a neural radiance field model is deployed for fine-grained reconstruction. The outcomes from the SFM step are fed into the neural radiance field model, which is adept at generating detailed and realistic reconstructions. The neural network’s capacity for iterative optimization allows the enhancement of the initial coarse model, leading to high-fidelity 3D models of potted plants. This hybrid methodology not only expedites the initial capture of the scene’s layout but also capitalizes on the neural network’s detailed rendering capabilities to refine the model.

### 2.3. Coordinate Correction of Point Cloud

In the realm of 3D modeling, particularly for potted plants, establishing the proportional relationship between the reconstructed model and the actual size is essential. This adjustment is facilitated by the use of a calibrated square, which serves as a reference object. Specifically, a square with a known side length of 0.03 m was included in the scene during the data capture process. The calibrated square enables the calculation of a proportionality factor, which is derived from the ratio of the square’s dimensions in the model to its actual dimensions. This factor is then applied to the entire point cloud model, thereby correcting the scale and ensuring that the model’s dimensions are proportional to those of the real potted plant. The specific formula is shown in Equation (2):

(2) $$K=\frac{H_{\mathrm{real}}}{H_{\mathrm{model}}}.$$

where $H_{\mathrm{real}}$ represents the actual physical length of the square grid used for calibration, and $H_{\mathrm{model}}$ denotes the corresponding length of the square as measured within the 3D model.

## 3. Stem and Leaf Segmentation

The assessment of potted plant phenotypic parameters is inherently intertwined with genetic analysis. When planted under the same environmental conditions, their external characteristics may vary due to genetic differences between individuals. The interception height of sunlight is determined by structural parameters, including the leaf size, stem diameter, and total plant height. These characteristics influence the competition for, and absorption of, light, which subsequently affects the growth patterns [30]. Before engaging in phenotyping, it is imperative to precisely segment the plant’s morphological structure [31]. A potted plant’s point cloud can be methodically dissected into three principal components: background elimination, the segregation of stems and leaf crowns, and the detailed segmentation of individual leaves.

### 3.1. Background Removal

The reconstructed model of the potted plant included point clouds from both the flower pot and the background. To augment the efficiency of the phenotyping process, extraneous point clouds were eliminated, thereby reducing the computational load. This was achieved through an initial phase of coarse background removal; the results of the filtering process are shown in Figure 4b.

The direct filtering process separates the potted plants from the background. Initially, this process establishes the base point of the potted plants, defined as the z-axis direction. The z-axis direction threshold is determined, and points within the specified threshold range are classified as parts of the potted plant. The specific formula is shown in Equation (3):

(3) $$Z_{\min}<Z_{i}<Z_{\max}.$$

The z-coordinate of the point cloud represents the vertical distance of potted plants. The minimum z-coordinate value is represented as $Z_{\min}$ corresponding to the position of the plant point cloud at the bottom of the stem. The maximum z-coordinate value is represented as $Z_{\max}$ indicating the position of the top of the plant point cloud leaves. Statistical filtering to eliminate spatially distant outliers helps reduce noise points at the edges of potted plant leaves.

Traditional segmentation algorithms for extracting stems have often relied on the random sampling consensus algorithm (RANSAC) [32]. However, the results are not very good when dealing with species that exhibit obvious stem curvature. This paper introduces an innovative methodology for the automatic classification, filtering, and segmentation of point cloud data from potted plants. The proposed algorithm is anchored in physical structural principles and is designed to execute these tasks autonomously, eliminating the requirement for manual oversight. The segmentation algorithm described employs a path-based approach to classify and segment the organs of a potted plant, utilizing the point cloud data from the stem and leaves as a reference. As shown in Figure 4c, the core concept of this path structure algorithm [33] is to first identify the primary path or the main stem component. It then differentiates the stem and leaf portions through the use of Boolean mask identification techniques. The retrieval of leaf segments is facilitated through a backtracking methodology, which involves each point in the dataset being systematically traced back a predetermined number of steps, following the path towards the root node. Concurrently, this method enables the identification of leaf segments, while simultaneously filtering out outlier points that reside at the periphery of the leaf edges.

**Figure 5:**
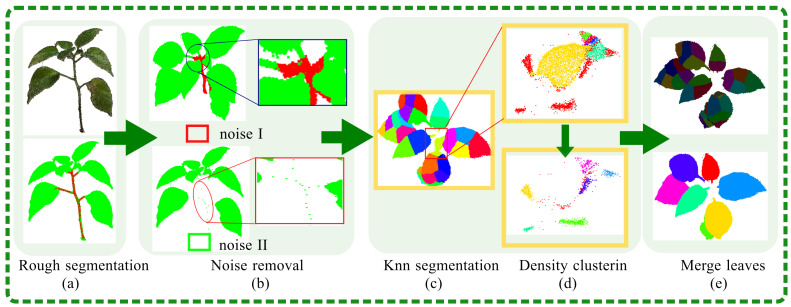
Specific methods for stem and leaf segmentation.The red color at the beginning represents the stem, and the green color represents the leaves. The colors in the middle of each leaf represent the fragmented pieces after segmentation. The subsequent colors correspond to the complete leaves formed by merging the fragments.

The presence of certain points that do not originate from the terminal portion of the leaf and do not recede a fixed number of steps can lead to an incomplete representation of the stem section. To maintain the integrity of the structure, it is essential to employ a dual approach of backward processing and gap-filling. This manuscript introduces a two-tiered segmentation procedure, which includes both coarse and fine segmentation stages, specifically designed for the path structure of potted plants. The coarse segmentation phase provides an initial rough delineation of the structure, while the fine segmentation phase refines this further to capture intricate details and ensure that the leaf and stem are accurately represented. The coarse segmentation process of the path structure is delineated as follows: (1) Triangular Mesh Creation: The initial step involves the construction of a triangular mesh object that encapsulates the plane within a 3D spatial framework. (2) Point Cloud Model Import: Subsequently, the reconstructed point cloud model of the potted plant is integrated into the triangular mesh, setting the stage for further processing. (3) Voxelization: To enhance computational efficiency, the point cloud model is voxelized within the triangular mesh. This transformation into a voxel grid facilitates quicker manipulation and analysis. (4) Root Node Identification: The voxel possessing the minimum Z-axis coordinate is identified and designated as the root node, providing an anchor point for subsequent analyses. (5) Path Analysis: A path analysis algorithm is then deployed on the voxel model to discern the primary stem of the potted plant, which is crucial for the structural analysis. (6) Path Mask Inversion: The inverse of the path mask is utilized to segregate points that do not align with the main path. This step is instrumental in differentiating between the stem and leaf. (7) Noise Removal: The outcomes of the coarse segmentation are presented in Figure 5a, where it is observable that the stem section still contains sparse noise points post-segmentation. These are eliminated through a neighborhood search algorithm that specifies a search radius and a threshold for point filtering. The algorithm is designed to remove isolated points or point clusters that have a neighborhood count below a predefined value, thereby classifying them as noise. (8) Further Segmentation Requirement: As illustrated in Figure 5b, the segmentation result for the newly sprouted area in the central region is poor, with leaves and stems being mixed.

The k-means clustering method [34] is utilized for the segmentation of point clouds, as demonstrated in Figure 5c. This approach effectively distinguishes various parts, yet challenges remain in segmenting the middle section of tender leaves. Specifically, stems and leaves enclosed within the same bounding box are often incorrectly segmented and merged into a single color, with different parts of the stems and leaves connected to the same region. To address these issues, particularly the difficult task of segmenting the staggered stems and leaves, this paper employs the DBSCAN algorithm [35]. This algorithm uses density as the basis for classification, enabling fine segmentation and improving the accuracy of potted plant organ segmentation. The density-based spatial clustering of applications with noise (DBSCAN) algorithm segments data by identifying dense regions connected by areas of lower density. The DBSCAN algorithm classifies points based on their local density. A point, is considered a core object if it has a sufficient number of neighboring points within a specified radius that meet a minimum density threshold. This radius is known as the ϵ-neighborhood (or ϵ-ball), and the minimum number of points required is the density criterion. Once a core object has been identified, the algorithm extends the cluster by including all direct density-reachable points from the core object. This process continues iteratively for all points within the cluster’s reach until no more points can be added to any clusters. Points that do not meet the criteria to belong to any cluster are classified as outliers or noise, thereby completing the segmentation process. The initial outcomes of the segmentation are presented in Figure 5d, where it is evident that the DBSCAN algorithm effectively resolved the previously misclassified areas. The stem and leaf portions are now accurately segregated. The stem is indicated by the red region, whereas the remaining regions are colored to represent the different leaf sections. Following the initial segmentation, a subsequent round of segmentation was performed on the obtained results, as depicted in Figure 5d. The DBSCAN algorithm applied a unique color-coding system to distinguish between the various segments of the stem. In parallel, the leaf section was also clearly differentiated, culminating in a comprehensive segmentation of the intricate stem and leaf architecture.

Following the clustering of leaf-enclosing boxes in the previous step, the contact and overlap areas between adjacent enclosing boxes can be computed. This calculation automatically assesses whether the threshold conditions for merging are met, thereby allowing the consolidation of fragmented leaf parts into a single, unified segment. The results of this process are illustrated in Figure 5e, which demonstrates the completion of the segmentation for individual leaves within the canopy. This approach ensures that the segmentation of each leaf is conducted with precision, facilitating a more accurate representation of the leaf’s structure and morphology.

### 3.2. Parameter Extraction for Potted Plants

**Figure 6:**
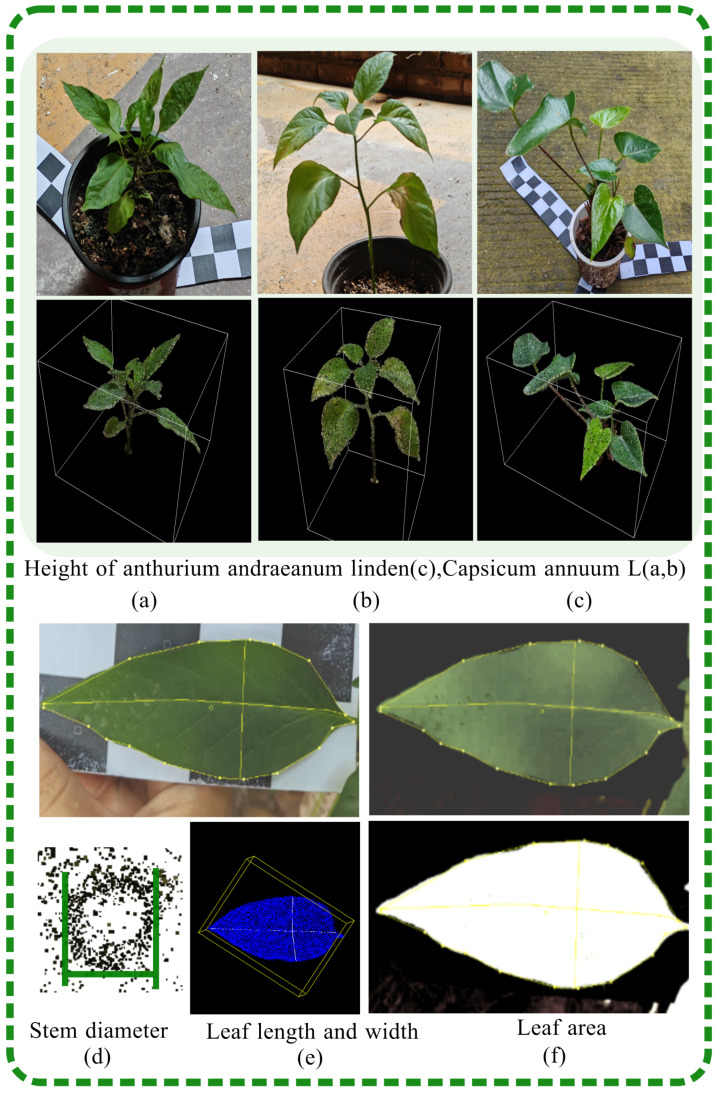
Schematic diagram of phenotype parameter measurement.

The three principal traits that define plant morphology are the leaf area, plant height, and stem thickness. Conventional methods of morphology research, which rely on manual measurement of these attributes, are not only inefficient but also susceptible to inaccuracies. In this study, we reconstructed and segmented the point cloud model of potted plants, subsequently extracting the structural parameters directly from the point cloud data. The implementation of a 3D point-cloud-based phenotype extraction method was shown to significantly enhance the accuracy of these measurements. The leaf, being the primary organ for photosynthesis, gas exchange, and transpiration, is crucial for potted plant growth and metabolism and is a key subject in phenotyping research [36]. Traditional methods of measuring leaf area, including manual and digital-technology-based techniques, have been improved but still face challenges in the context of complex shading and overlapping. This results in incomplete and scattered leaf point clouds, affecting the accuracy of parameter calculation. To address these issues, this study used reconstructed point clouds of potted plants for area calculation, as shown in Figure 6f. The leaf area was determined utilizing a greedy projection algorithm [37], which facilitated the triangulation of the leaves. This process generates a series of triangles, each encapsulating the original 3D point cloud data. The vertices of these triangles are calculated to ascertain the lengths of the three edges, the area of an individual triangle is calculated using Heron’s formula, and the areas of all triangles are summed to derive the total leaf area. In the academic context, measuring plant height is a critical parameter for assessing plant growth status, managing plant populations, and advancing plant breeding research. Various methodologies are commonly employed, including direct measurement, angular measurement, and acoustic wave measurement. However, the accuracy of certain methods can be inconsistent, and there is often a need for improvement in the speed of realization. The height of a potted plant is defined as the vertical distance from the base of the plant to the highest point of its foliage. As shown in Figure 5a–c, the reconstructed point clouds of *Anthurium andraeanum Linden* and *Capsicum annuum* L. are enclosed within a bounding box. The height can be measured by determining the difference in height from the apex of the segmented stem’s foliage to the base. The formula for calculating plant height (HH) can be expressed as follows:

(4) $$H=\max\left(Z_{\mathrm{plant}}\right)-\min\left(Z_{\mathrm{plant}}\right).$$

where $\max(Z_{\mathrm{plant}})$ denotes the maximum z-coordinate value of the plant’s point cloud, indicating the topmost point of the plant, and $\min(Z_{\mathrm{plant}})$ denotes the minimum z-coordinate value, indicating the base of the plant.

The capsule box is used for extracting parameters in phenotype analysis, providing enclosed spaces for discrete points and allowing for the simplification of complex point cloud data into geometric approximations. After the segmentation process, the leaves are retained in the global coordinate system, ensuring that their spatial relationships and positions are preserved, as shown in Figure 6e. We adopted an oriented bounding box (OBB) obtained through principal component analysis (PCA). The direction of the OBB is not limited to the axis and is suitable for point cloud data. This direction ensures that the boundary objects are concentrated in space, closer to the actual shape of the leaves. After constructing the OBB, the point cloud of the leaves is transformed into a coordinate system defined by the OBB. In this system, the maximum and minimum distances of points in each axis direction are calculated. The measurement of leaf length is a critical parameter in assessing the nutritional status and growth rate of potted plants, as well as for informing agricultural management strategies. Traditional methods for measuring leaf length include direct measurement with rulers, laser ranging, and image analysis. However, these methods can be time-consuming and may not be as precise as needed for detailed phenotyping. The two farthest points in the leaf point cloud space are defined as the endpoints of the leaf length.

﻿Stem thickness is a critical indicator of potted plant health and growth, reflecting the plant’s capacity to accumulate biomass, store nutrients, and maintain structural integrity against collapse. In this study, we employed a precise method to measure stem thickness, which is essential for accurate phenotyping. Traditional methods, such as the use of soft or vernier calipers, are time-consuming and often lack the required precision. To standardize our measurements, we selected a fixed position 0.05 m above the pot surface as the measurement site for stem thickness. The measurement procedure involved calculating the difference between the two furthest points in the horizontal direction of the stem at the defined site, as illustrated in Figure 6d.

To evaluate the effectiveness of the 3D reconstruction method for determining the phenotypic parameters of potted plants, the accuracy of the reconstruction was empirically tested by manually measuring the true values of various parameters. The specific phenotypic parameters included the leaf area, plant height, leaf width, leaf length, and stem thickness. For leaf area measurement, a calibrated paper tape of known dimensions was utilized as a backdrop for capturing an image of the leaf. Subsequent image processing was conducted to facilitate the computation of the leaf’s area, as shown in Figure 6f. Initially, the chromatic components were adjusted by enhancing the green channel’s luminance, while diminishing that of the red and blue channels, thereby isolating the leaf from the complex background. Following this, the image was transformed into a grayscale format to enable the calculation of the leaf’s area. The determination of potted plant height was conducted by measuring from the soil surface to the apex of the stem and leaves. A ruler was positioned vertically adjacent to the main stem, ensuring its base was aligned with the soil surface. The measurement was taken from this reference point to the highest point of the foliage, with the observer’s line of sight kept parallel to the ruler’s scale for accuracy. Each measurement was recorded, and this process was repeated at multiple points along the potted plant to obtain a series of height values; the average of these measurements was then computed to determine the mean height. For leaf width and length, the dimensions were determined by taking multiple measurements with a tape measure and calculating the average distance to ensure precision. For stem thickness, the diameter of the stem was recorded by selecting the widest point on the main stem and aligning a vernier caliper parallel to it. The average value was derived from several such measurements to accurately determine the stem’s thickness.

### 3.3. Calculation of Assessment Indicators

Stem and leaf segmentation was performed on the reconstructed potted plant model using the proposed point cloud segmentation methodology. To distinguish between the stem and the leaves in the transitional region where stems and leaves meet—often a challenging area due to the complexity of the structure—we employed specific techniques. The efficacy of the segmentation was assessed by quantifying the number of successfully segmented leaf point clouds.

(5) $$R_{l}=\left(\frac{N_{l}}{N}\right)\times 100\%.$$

Let $N_{l}$ represent the count of plant leaf point clouds after segmentation, and let *N* denote the total count of plant point clouds before the segmentation process. The segmentation rate of plant leaf point clouds is signified by $R_{l}$; this rate provides a quantitative measure of the segmentation method’s performance, indicating the accuracy and reliability with which the leaves have been separated from the stem and other parts of the potted plant. The purpose of leaf segmentation is to distinguish leaves within the plant canopy and to evaluate the segmentation effect based on the total number of clustered leaf point clouds. The variable $N_{S}$ represents the total number of point clouds in the canopy leaves, while $N_{l}$ represents the total number of point clouds in the canopy before segmentation. The segmentation rate is represented as $R_{S}$, which is defined in Equation (6).

(6) $$R_{S}=\left(\frac{N_{S}}{N_{l}}\right)\times 100\%.$$

In the domain of plant science research, the extraction of phenotypic parameters is essential for monitoring the growth of potted plants and serves as a critical benchmark. This study aimed to evaluate the accuracy of an extraction method by comparing the shape parameters derived from a 3D reconstruction of *Capsicum annuum* L. with those obtained through manual measurements. The phenotypic parameters under consideration included the plant height, stem thickness, and leaf area.

To evaluate the accuracy of the methodological measurements for extracting phenotypic parameters of potted plants, the (R^2^) statistical metric was utilized. (R^2^): This statistic indicates the proportion of the variance in a dependent variable that is predictable from the independent variables. It is calculated using the following formula:

(7) $$R^{2}=1-\frac{\mathrm{SSE}}{\mathrm{SST}}.$$

where SSE is the sum of squares due to error, and SST is the total sum of squares.

## 4. Results

### 4.1. Result of 3D Reconstruction

**Figure 7:**
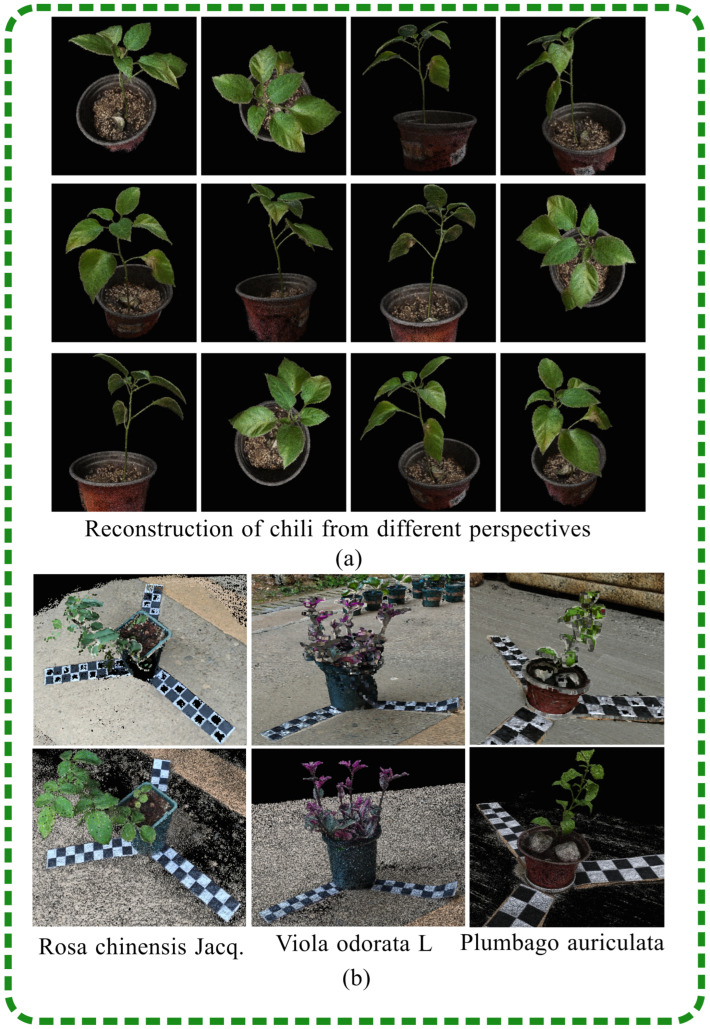
Three-Dimensional Reconstruction Results: As shown in (**a**), the reconstructed point clouds of *Capsicum annuum* L., captured from various angles, exhibit excellent brightness, sharp edges, and realistic material properties. (**b**) displays the reconstruction results for *Rosa chinensis* Jacq., *Viola odorata* L. and *Plumbago auriculata.*, obtained using the SFM-MVS and SFM-NeRF methods, presented in the first and second rows, respectively. The point clouds reconstructed by the SFM-NeRF method generally lack noise at the target edges and show no missing leaves on roses.

The point cloud of the *Capsicum annuum* L. plant, generated using the SFM-NeRF method, not only includes high-fidelity 3D data but also incorporates color attributes derived from the original image. As shown in Figure 7a, our dense point cloud model clearly represents the geometric structure and fine texture of the entire *Capsicum annuum* L. The 3D point cloud data of the *Capsicum annuum* L. were approximately 24 MB, providing a solid foundation for subsequent 3D analysis tasks.

As shown in Figure 7b, the point cloud models of *Rosa chinensis Jacq., Viola odorata* L. and *Plumbago auriculata* generated by the SFM-NeRF method (second row) were superior to those generated by the traditional SFM-MVS method (first row). The SFM-MVS method’s point cloud model captures geometry with fewer details and sparse regions, while our model offers a more complete and detailed surface reconstruction. The SFM-MVS method required approximately 2 h for reconstruction, whereas our model, which generated point cloud models without defects or gaps and with accurate color representation, reduced the reconstruction time to 1 h.

**Figure 8:**
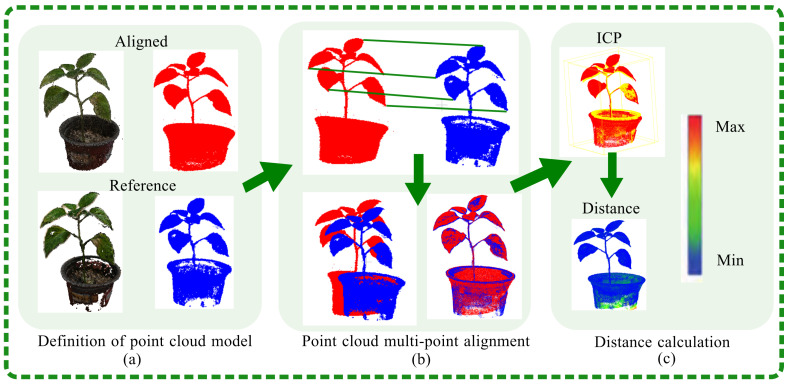
This figure presents a comparative analysis of point cloud reconstructions using the SFM-NeRF and SFM-MVS methods, as facilitated by CloudCompare. (**a**) displays the reference point clouds for *Capsicum annuum* L. reconstruction, where red represents the SFM-NeRF method and blue represents the SFM-MVS method. (**b**) shows the process of selecting distant point pairs for correspondence and subsequent alignment. (**c**) depicts the distance calculation for alignment accuracy assessment.

A comparative analysis between the 3D models generated by the SFM-MVS technique and those produced by the approach detailed in this paper was conducted using CloudCompare. In Figure 8a, the point cloud models were aligned by importing both the comparison model and the reference model, with distinct color settings applied to differentiate the point clouds. In Figure 8b, the align tool selected three or more corresponding point pairs from the two point clouds for alignment. In Figure 8c, precise alignment of the models was accomplished using the iterative closest point (ICP) fine alignment tool. The degree of overlap, typically quantified by distance, was used to measure the alignment accuracy. The Euclidean distance $D_{i}$ between two points in 3D space is calculated as

(8) $$D_{i}=\sqrt{{\left(x_{C}-x_{r}\right)}^{2}+{\left(y_{C}-y_{r}\right)}^{2}+{\left(z_{C}-z_{r}\right)}^{2}}.$$

The average distance *R* between the point clouds is then determined by

(9) $$R=\sqrt{\frac{\sum_{i=1}^{n}D_{i}^{2}}{n}}.$$

**Figure 9:**
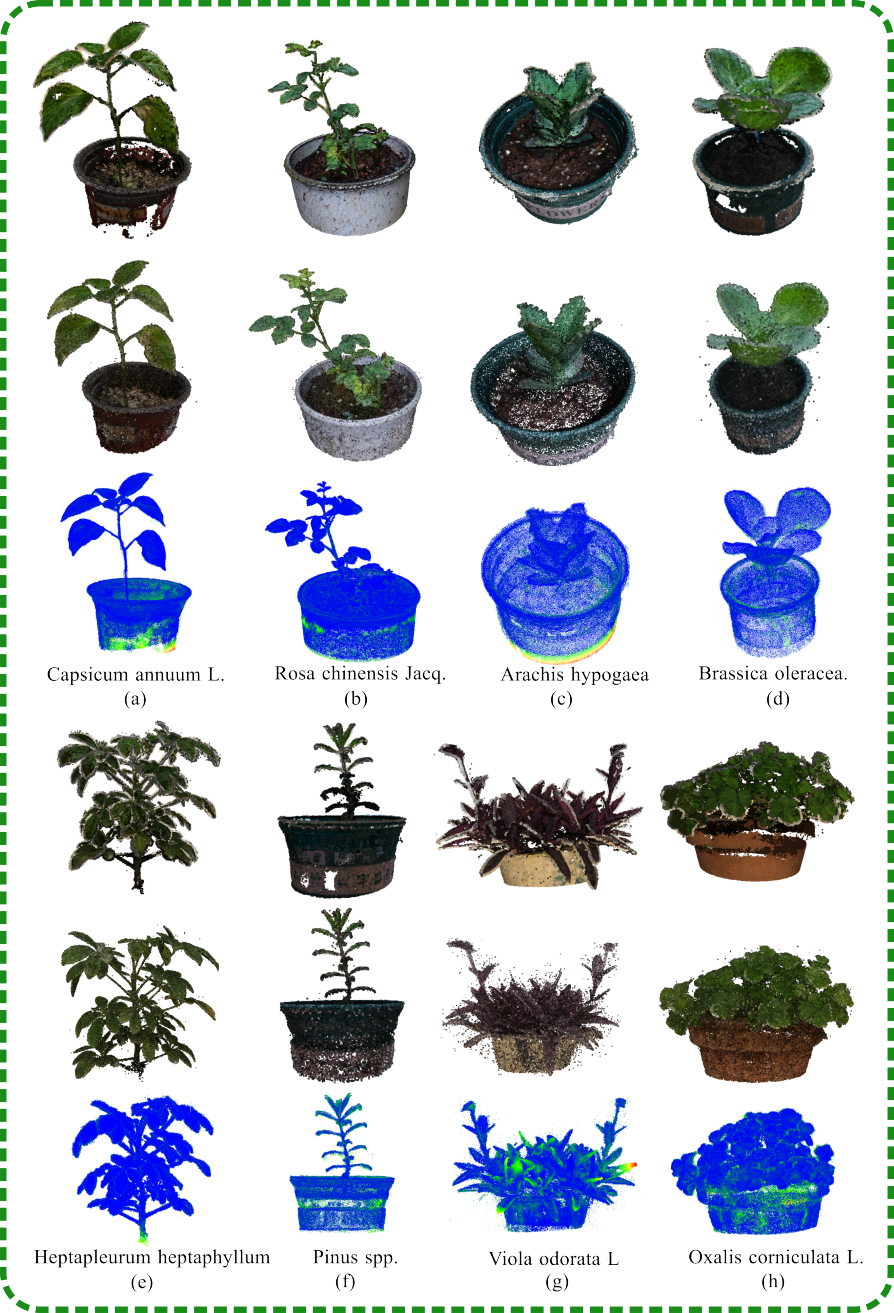
This figure presents a comparative analysis of the reconstruction robustness of the SFM-NeRF and SFM-MVS methods on various potted plants. The reconstruction quality was assessed by calculating the distances between corresponding points within the point clouds generated by each method. The first and fourth rows of the figure display the SFM-MVS reconstructions, which include *Capsicum annuum* L., *Rosa chinensis* Jacq., *Arachis hypogaea* and *Brassica oleracea*. The second and fifth rows showcase the SFM-NeRF reconstructions, with *Heptapleurum heptaphyllum*, *Pinus* spp., *Viola odorata* L. and *Oxalis corniculata* L. The blue areas in rows 2 and 5 indicate regions where the reconstruction quality of the two methods was similar, while the red areas denote regions with significant differences. The SFM-NeRF method demonstrated a reconstruction quality that was comparable to the SFM-MVS method.

This study employed a visualization method to assess the accuracy of the NeRF reconstructions. The process involved measuring the distance between two sets of point clouds: one from the SFM-MVS reconstruction, which served as a baseline reference, and the other from the NeRF approach. ﻿ To evaluate the versatility of the SFM-NeRF modeling, a range of potted plants were assessed, including *Ribes uva-crispa*, *Echinops ritro*, *Viola odorata* L., *Oxalis corniculata* L., *Capsicum annuum* L., *Rosa chinensis* Jacq., *Arachis hypogaea* and *Brassica oleracea*. As shown in Figure 9, blue areas in the visualization indicate high reconstruction quality, while the red areas represent regions of lower quality.

﻿ The divergence in results between the two methodologies was due to the NeRF model’s capability for intricate modeling. The NeRF method characterized the model through a continuous 5D vector function. This was achieved by outputting color information and volume density along the viewing direction and integrating these attributes through a classical volumetric rendering algorithm. The NeRF model’s reconstruction process involved continuous integration of color and density data. This integration was part of a larger optimization process where parameters were fine-tuned to minimize the error between actual observations and the model’s predictions.

#### 4.1.1. Effect of Algorithm Parameters on Leaf Segmentation Accuracy

**Figure 10:**
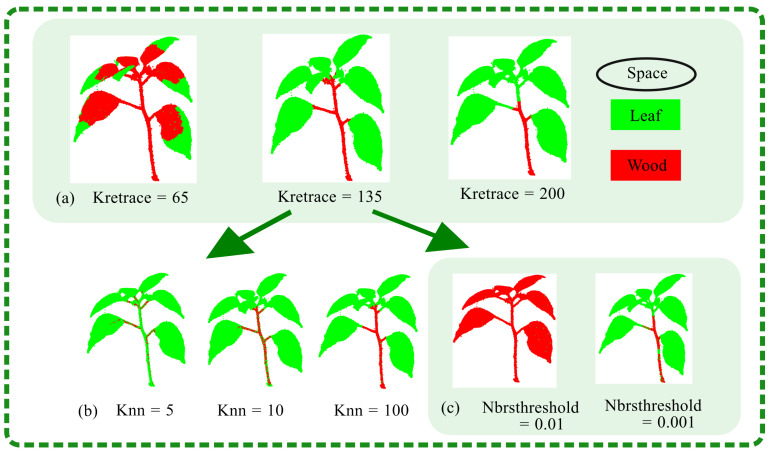
Path analysis results under different parameters.

The effectiveness of path-based segmentation for distinguishing stems and leaves in point clouds is influenced by several key parameters: the number of gaps to be filled in the path, denoted as $K_{nn}$, the number of steps to backtrack from the endpoint for each node in the graph, denoted as $K_{retrace}$, and the maximum distance of valid neighboring points used to fill the path gaps, denoted as $Nbrs_{threshold}$. As illustrated in Figure 10b, $K_{nn}$ affects the size of the stem region within the point cloud. An increased $K_{nn}$ value allows for the inclusion of more neighboring points, enhancing the gap-filling effect within the stem and improving the accuracy of stem identification. However, a higher $K_{nn}$ value also leads to greater memory consumption, potentially reducing the algorithm’s efficiency. As illustrated in Figure 10a, the parameter $K_{retrace}$ is primarily used to determine the leaf regions in the point cloud. If $K_{retrace}$ is set too low, the number of backtracking steps is insufficient to generate the complete leaf segment. Conversely, if $K_{retrace}$ is too high, this results in excessive backtracking, which may misclassify parts of the stem as leaves. As illustrated in Figure 10c, $Nbrs_{threshold}$ is the main factor affecting the overall point cloud’s inter-point distances. If $Nbrs_{threshold}$ is set too high, this can reduce the accuracy of the segmentation, as too many neighboring points may erroneously fill the gaps in the point cloud, leading to an inaccurate representation of the stem. On the other hand, if $Nbrs_{threshold}$ is too low, this may result in inadequate filling of the stem gaps, failing to meet the segmentation requirements for accurate potted plant modeling.

The selection of $K_{nn}=5$ enhanced the segmentation efficiency but results in insufficient point cloud connectivity, leading to incomplete stem path filling and misclassification of point clouds as leaves. Therefore, increasing $K_{nn}$ is recommended for better performance. For $Nbrs_{threshold}=0.01$, the connectivity between point clouds was excessive, causing paths to extend into leaf areas and be incorrectly identified as stems. Adjusting $Nbrs_{threshold}$ to a lower value is necessary for improved accuracy. Experimentation indicated that $K_{retrace}=65$ is too low, causing leaves to be misclassified as stems, while $K_{retrace}=200$ is too high, leading to the misclassification of stems as leaves. The optimal value for $K_{retrace}$ lies within the range of 65 to 200. In cases with minor leaf adhesion and occlusion, setting $K_{nn}=600$, $K_{retrace}=135$, and $Nbrs_{threshold}=0.003$ achieves effective segmentation.

#### 4.1.2. Analysis of Organ Segmentation Results

**Figure 11:**
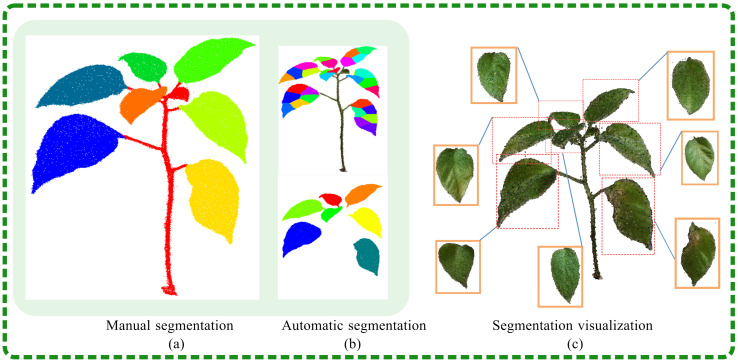
Comparison of actual manual segmentation and automatic segmentation results.The colored leaf on the left is the standard single leaf. The upper part of the middle section represents the fragmented leaf pieces after segmentation, while the lower part shows the successfully merged leaf.

In this study, the segmentation of potted plant organs was visualized, as shown in Figure 11c. Figure 11a displays manual segmentation, while Figure 11b displays the result of automatic segmentation. The algorithm segmented the point cloud model of *Capsicum annuum* L., with the number of leaves consistent with expectations. The segmentation evaluation results are detailed in Table 1, which reports the segmentation rates for stems and leaves, as well as for adherent leaves, with the highest values reaching 95.8% and 99.1%, respectively. These findings confirm that the segmentation algorithms could effectively extract the key phenotypic organs from the point cloud data.

### 4.2. Analysis of the Results of Calculating Phenotypic Parameters

**Figure 12:**
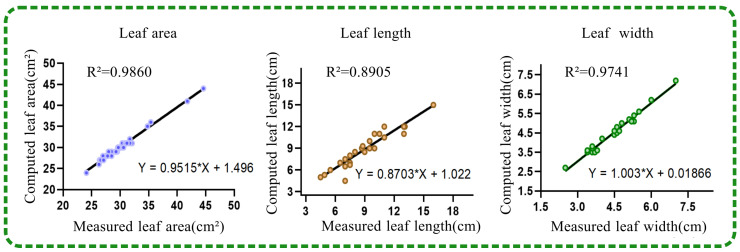
Comparison between measured and actual values of *Capsicum annuum* L. phenotype parameters.

By comparing the obtained phenotype parameters—leaf area, leaf length, and leaf width—with the manually measured values to calculate the accuracy of the results, Figure 12 present the linear equations between the calculated and measured values for leaf area, leaf length, and leaf width, respectively: Y = 0.9515X + 1.496, Y = 0.9515X + 1.496, Y = 0.8703X + 1.022, Y = 0.8703X + 1.022, and Y = 1.003X + 0.01866, Y = 1.003X + 0.01866. The corresponding $R^{2}$ values were 0.9860%, 0.8905%, and 0.9741%, respectively. The results demonstrate that the plant height and leaf area extracted using the method of this study had a high correlation with the manually measured values, thereby verifying the practicality and stability of the phenotypic parameter extraction method. Although the algorithm’s measured value for leaf width was close to the actual value, the relative error was higher compared to other phenotypic parameters, indicating that the method used in this paper still has room for improvement in leaf width measurement.

## 5. Discussion

### 5.1. Comparison of Three-Dimensional Reconstruction

Currently, three prominent methods for plant reconstruction are utilized: depth camera reconstruction, LiDAR-based scanner reconstruction, and SFM-MVS reconstruction using structured light. Depth cameras, known for their rapid measurement capabilities, capture depth information from potted plants but are susceptible to inaccuracies due to factors such as reflectivity, imaging distance, and ambient light. LiDAR-based point cloud reconstruction offers the benefits of uniformity and high precision, albeit at a typically high cost. In contrast, SFM-MVS reconstruction, which requires only a single RGB camera for data collection, has become a prevalent 3D reconstruction technique. This study, therefore, compared the SFM-NeRF-based reconstruction method with the SFM-MVS point cloud reconstruction method across metrics of accuracy, cost, data collection efficiency, and applicable scenarios. The environment for data collection significantly impacts plant reconstruction. To assess the robustness of the SFM-NeRF method, experiments were conducted both outdoors, where conditions included a gentle breeze and uneven lighting, and indoors, with no wind and even lighting. The experiments also varied whether a turntable was used and the placement of the calibration paper, conducting point cloud reconstruction on 22 plants. Table 2 presents a comparison of the reconstruction results between the SFM-NeRF and SFM-MVS methods under these different conditions. The last two columns of Table 2 display the reconstruction outcomes for the SFM-NeRF and SFM-MVS methods, respectively. When outdoor images were used as input (the first 13 rows of Table 2), the SFM-NeRF method demonstrated a higher reconstruction success rate than the SFM-MVS method. The SFM-MVS method was better suited for indoor image acquisition, requiring uniform lighting and being sensitive to wind disturbances. In contrast, the NeRF model could iteratively learn from input data, overcoming a certain degree of interference. Notably, the reconstruction of indoor *Orchidaceae* and outdoor *Capsicum annuum* L., as shown in the 8th and 9th rows of Table 2, failed despite the use of a turntable and adequate calibration. This error was attributed to the similarity in plant surface colors and the bottom calibration paper’s length being limited by the turntable size, leading to unclear changes in the angle of view during collection and subsequent failures in camera pose estimation. It was also found that only the bottom calibration paper was necessary, as it had to occupy a sufficient proportion of the image to highlight differences. Figure 6b offers a more intuitive view of the reconstructed point clouds for *Rosa chinensis Jacq, Viola odorata* L. and *Plumbago auriculata* using the SFM-NeRF method, which is relatively complete, with less noise at the edges and smoother surface textures. The lower part of Table 2 indicates that the SFM-MVS method had a higher reconstruction success rate with a fixed camera, reducing the flexibility in outdoor applications. The SFM-NeRF method, however, could ensure a high reconstruction success rate with a mobile camera, making it more practical. The SFM-MVS method required nearly 2 h for reconstruction, while the SFM-NeRF method reduced the reconstruction time to 1 h. The intuitive reason for this is evident in Figure 6b: the background potted plant behind the *Viola odorata* L. was also reconstructed, showing that the SFM-MVS method captured additional scene elements, whereas the SFM-NeRF focused solely on reconstructing the target plant. The potted plants listed in Table 2 were collected from the laboratory of Hunan Agricultural University.

### 5.2. Leaf and Stem Segmentation

For the segmentation of potted plants, traditional methods face some difficulties in finely separating stems and leaves, such as various means of clustering. This is because of the complex topology structure of a potted plant compared to *Zea mays*, *Sorghum bicolor,* and other crops. This conclusion is also verified in Figure 2, which shows that KNN is the classic segmentation method for segmenting potted plant leaves. We found the key to the means of clustering is how to determine suitable parameters. A model will separate parts of a leaf if the parameters are set too high. In contrast, some leaves may be in the same class, leading to missegmentation. To solve this, we introduced path analysis for clustering preprocessing. The clustering methods obtain the optimal number of clusters, a key parameter for clustering. Path analysis reduces the point clouds of the potted plant, making the topology simpler. The leaves of potted plants are different from the roots. As such, path analysis first separates the leaves, and the number of leaves is estimated; this number is an essential parameter for clustering. Thus, the result is a significant improvement using a given suitable parameter. Figure 5 is valid for this result. Figure 5a shows a rough segmentation with the process of path analysis. The isolated points are removed in Figure 5b to suppress noise. Figure 5c shows the path analysis process followed by the clustering process, which used KNN clustering and density clustering. Finally, the leaves were separated. As shown in Table 1, path analysis for segmentation was successful in all plants except for crassula. The top leaves of crassula are close to each other, so some leaves became a single leaf if the information is just taken from images, ignoring preknowledge. Artificial intelligence could solve this by analyzing preknowledge. In other words, path analysis is only helpful for segmentation if plant point clouds have good structural features.

### 5.3. The Analysis of the Phenotypic Parameters

The phenotypic parameters were highly accurate, as proven by the correlation between the measured results and the calculated values in Figure 12. Although the parameters of leaf area, leaf length, and leaf width were very close to the measured values, and the R values were 98.6%, 89.05%, and 97.41%, respectively, some slight errors were still specified for the leaf length. The reason for these slight errors is that there are always some indistinct points, because the edge of the leaf is unclear. To decrease this error, we have to manually measure the leaf many times and average it to decide the parameters. Artificial intelligence may be a good solution for this issue. Although the model could not achieve absolute accuracy, the calculated values of phenotypic parameters can help us in breeding and cultivating.

### 5.4. Future Work

This study demonstrated success across a variety of potted plants. To further strengthen and localize our findings, several avenues for future research are proposed. Firstly, we aim to collect imagery at various developmental stages of plants to elucidate the environmental influences on potted plant growth dynamics. Secondly, collaboration with botanical experts is envisioned to investigate the role of canopy leaf inclination on photosynthetic efficiency within different plant species. Thirdly, the deployment of cameras on mobile platforms for remote sensing will be explored to enhance data acquisition capabilities. Lastly, we plan to integrate novel LiDAR scanning technologies to assess the three-dimensional growth patterns of plant roots, thereby providing a comprehensive view of plant growth in diverse local conditions.

## 6. Conclusions

In this study, we developed a methodology that integrates NeRF and path segmentation algorithms for the 3D reconstruction and segmentation of stems and leaves. Potted plant image datasets were collected using both stationary and mobile cameras in various settings, both indoors and outdoors, to serve as the basis for model reconstruction. Following the segmentation of organs, phenotypic parameters were extracted. The analysis demonstrated a high correlation between the extracted measurements and actual metrics, indicating the efficacy of the methodology in accurately capturing potted plant phenotypes. The study also investigated the impact of chessboard pattern calibration on reconstruction accuracy. The method’s applicability was further assessed by reconstructing and validating it across different potted plant species. In summary, the proposed approach, which utilizes a low-cost setup, has the potential to significantly advance future research in phenotyping.

## Figures and Tables

**Table 1:** Statistics of stem and leaf segmentation and canopy leaf segmentation results.

Crop	Before	After	Prop.	Before	After	Prop.
	Stems	Stems	Stems	Bud	Bud	Bud
*Capsicum annuum* L.	321 K	306 K	0.953	306 K	299 K	0.977
*Rosa chinensis* Jacq.	52 K	50 K	0.958	50 K	49 K	0.983
*Plumbago auriculata*	59 K	51 K	0.865	51 K	48 K	0.928
*Anthurium andraeanum* Linden	149 K	132 K	0.884	132 K	131 K	0.995
*Crassula mesembryanthoides*	10 K	9.7 K	0.895	/	/	/
*Capsicum annuum* L.	295 K	270 K	0.916	270 K	262 K	0.970
*Abutilon theophrasti*	942 K	884 K	0.938	884 K	876 K	0.991

**Table 2:** Experimental investigation into the impact on the reconstruction quality of potted plants. The check mark indicates the measures taken, while the last two check and cross marks indicate whether the reconstruction was successful.

Crop Variety	Outdoor	Indoor	Turntable	Side Calibration	Bottom Calibration	SFM-NeRF	SFM-MVS
*Chrysanthemum morifolium*	√				√	√	×
*Capsicum annuum L.2*	√			√	√	√	√
*Rosa chinensis Jacq.*	√			√	√	√	√
*Viola odorata* L.	√			√	√	√	√
*Anthurium Andraeanum Linden*	√			√	√	√	√
*Brassica oleracea*	√			√	√	√	√
*Crassula Mesembryanthoides*	√			√	√	√	√
*Rosa chinensis Jacq.2*	√			√	√	√	√
*Capsicum annuum L.3*	√			√	√	√	×
*Abutilon Theophrasti*	√			√	√	√	×
*Hydrangea spp.*	√			√	√	√	×
*Bryophyllum Pinnatum*	√		√	√	√	√	√
*Capsicum annuum L.*	√		√	√	√	×	×
*Orchidaceae*		√	√	√	√	×	×
*Tagetes erecta*		√	√	√	√	√	×
*Plumbago auriculata2*		√	√	√	√	√	×
*Viola odorata L.2*		√	√		√	√	√
*Oxalis corniculata*		√	√		√	√	√
*Heptapleurum Heptaphyllum*		√	√	√	√	√	√
*Viola odorata L.2*		√	√		√	√	√
*Plumbago auriculata*		√		√	√	√	√

## Data Availability

Data are contained within the article.
